# Practitioner Self-Reports of Adherence to Evidence-Based Practices for Autistic Youth: Psychometric Properties and Association with Youth Outcomes in a Multiple Baseline Study

**DOI:** 10.1007/s41252-026-00475-3

**Published:** 2026-01-23

**Authors:** Kashia A. Rosenau, Jeffrey J. Wood, Karen S. Wood, Jolie Straus, Bryce D. McLeod

**Affiliations:** 1 David Geffen School of Medicine, University of California, Los Angeles, USA; 2 Department of Education, University of California, Los Angeles, USA; 3 Department of Psychology, Virginia Commonwealth University, Richmond, USA

**Keywords:** Treatment adherence, CBT, Autism, Self-report

## Abstract

**Objectives:**

Practitioner ratings of their adherence to evidence-based psychotherapy or counseling practices in working with autistic youth could be an efficient method for tracking the success of efforts to implement effective practices in community behavioral health service settings. Tools of this sort that generalize across multiple evidence-based practices (EBPs) have not been developed to date. In this study, a practitioner self-report measure of adherence to EBPs for autistic youth was developed and evaluated.

**Methods:**

A multiple baseline study of seven behavioral health practitioners was conducted to determine if internet-based self-directed training on Modular EBPs for Youth on the Autism Spectrum (MEYA; meya.ucla.edu), a free internet-based practitioner training app, would facilitate an increase in clinician adherence to EBP techniques such as cognitive behavioral therapy and behavioral intervention practices. Practitioners made self-ratings on the MEYA Integrity Scale-Practitioner Version (MEYA-IS-PV). Expert coders unaware of phase or study hypotheses also rated the therapists’ adherence to EBPs for each session. Caregivers rated youth outcomes at each session on the Youth Top Problems scale.

**Results:**

Linear mixed models showed that MEYA-IS-PV scores corresponded to expert ratings of adherence, increased following the onset of training in EBPs, and predicted improvements in youth behavioral health. Visual analysis of the multiple baseline data confirmed that 5 of 7 practitioners increased their self-reported adherence in EBPs in varying magnitudes once training began.

**Conclusions:**

Self-ratings of adherence are promising for examining adherence to EBPs in psychotherapy or counseling for autistic youth.

Measures of treatment adherence to specific outpatient behavioral health practices—defined as the degree to which clinicians deliver a treatment as designed (Allen et al., 2012)—are needed to ascertain the success of efforts to implement evidence-based programs (EBPs), such as cognitive-behavioral therapy (CBT) and behavioral interventions (BIMcLeod et al., 2023; Wood et al., 2015a). While much research attention has been paid to the ascertainment of EBPs for autistic individuals in efficacy studies conducted in research settings with recruited samples (Weston et al., 2016), very few studies have examined the effectiveness of these EBPs in service settings with their typical clientele of autistic youth (i.e., referred for mental health needs such as depression, anxiety, or disruptive behavior). Relatedly, little research has been done on factors impacting the successful implementation of EBPs like CBT and BI in these settings. Among the critical measurement tools needed to examine the success of efforts to implement EBPs in community care settings are instruments assessing adherence to EBPs (McLeod et al., 2013).

Treatment adherence is commonly assessed with observational ratings of recordings of behavioral health sessions or practitioner self-ratings of specific intervention techniques (McLeod et al., 2023). These approaches each have advantages and disadvantages. Observational ratings can minimize the chance of rater biases and may overcome the impact of clinician memory on their ratings. Self-reports are cost-effective, efficient, and can tap into both practitioners’ observed behavior as well as the purpose or meaning of their practices (e.g., is playing a game during a session an in-session reward, a form of behavioral momentum, role-play, or an autism-related treatment adaptation to encourage engagement on concurrent therapeutic discussions). In many service settings, the need for confidentiality or other systemic constraints, as well as practitioner and family comfort, may also necessitate reliance on measurement techniques that do not rely on session recordings. Thus, an important question is whether practitioner self-reports display adequate score validity to serve as an alternative to observational ratings (McLeod et al., 2023).

Several EBPs for autistic youth have emerged over the past two decades, with evidence of efficacy for several specific (e.g., behavioral contracting) and more general (e.g., CBT) practices across a range of treatment outcomes (e.g., mental health, social engagement, self-care skills; Hume et al., 2021). As noted above, in general, the effectiveness of these practices has not been examined in community settings such as community mental health centers and implementation measurement tools will be necessary to help examine the success of their adoption in such settings and the impact of the practices on youth outcomes in order to assess the rationale for the implementation of specific EBPs in community psychotherapy and counseling practice.

Recently, a compendium of EBPs for autistic youth in psychotherapy or counseling has been developed in a series of efficacy trials (e.g., Storch et al., 2013, 2015; Wood et al., 2009, 2015b, 2020, 2022) that have led to the development of a free/open-access training and implementation support platform entitled Modular EBPs for Youth on the Autism Spectrum (MEYA; Wood et al., 2024). This platform, in turn, has been assessed for its impact on practitioner adherence to the specific EBPs in this compendium through expert ratings of session content in a multiple baseline study (Wood et al., 2024). The expert ratings measure, MEYA-FS, was first developed and refined in a psychometric study of archival session data from the efficacy trials (McLeod et al., 2022a) with a sample of 77 autistic youth ages 6 to 13 years old. The psychometric study found evidence of score reliability (i.e., inter-rater agreement), discriminant validity, and predictive validity for the MEYA-FS Adherence and Competence subscales. The multiple baseline study results showed that following the onset of MEYA-based training, most practitioners measurably increased their adherence and competence scores on the MEYA-FS, offering evidence of criterion-related validity (Wood et al., 2024).

While the expert rating measure developed for MEYA is a promising option for future effectiveness and implementation research on EBPs for autistic youth in psychotherapy or counseling, practitioner self-ratings of adherence could be an important tool that may be needed for some community care settings in which practicality or participant preference make session recordings untenable (Dear et al., 2024). The present study is a secondary data analysis of practitioner self-reports of adherence to MEYA practices from the same multiple baseline study that reported on expert ratings of fidelity noted above (Wood et al., 2024). In the present study, a practitioner self-report adherence measure—the MEYA Integrity Scale-Practitioner Version (MEYA-IS-PV)—was assessed for inter-item correlations, convergence with expert ratings of adherence, responsiveness to MEYA training, and association with youth outcomes as a multifaceted initial assessment of its psychometric properties.

## Methods

This study is one component of a multifaceted project funded by the National Institute of Mental Health (1R34MH110591) that supported the development of the MEYA training platform and the initial evaluation of its success in supporting practitioner fidelity and youth outcomes. MEYA, the expert rating measure of fidelity developed for it (MEYA-FS), and the participants in this study are described in detail in McLeod et al. (2022a) and Wood et al. (2024). These study elements are summarized below, with additional detail provided on the practitioner self-rating measure, MEYA Integrity Scale-Practitioner Version (MEYA-IS-PV), which is the focus of the present study.

### Participants

Seven practitioners conducting psychotherapy or counseling with children or teens on the autism spectrum participated in this study. All participants resided in the USA, and their eligibility was determined through an initial phone screening. Eleven practitioners initially expressed interest, but three withdrew due to difficulty identifying eligible child participants, and one chose not to participate, leaving seven who completed the study.

Practitioners were recruited through organizations such as the Autism Speaks Autism Treatment Network, medical centers, parent support groups, and schools. Interested practitioners initiated contact with the study coordinator. Following written consent, practitioners provided study details to eligible families. Informed consent was obtained from both practitioners and parents, while children assented after receiving a full explanation of the study. Participating children scored above a *T*-score of 68 on the SRS-2, reflecting moderate to extensive autism-related challenges (Constantino & Gruber, 2012), and above the first percentile on the WISC-V Vocabulary subtest. Practitioners received $250 in gift cards for participation, while families received $100 split equally between parents and children. The study was conducted remotely through the university affiliated with the lead author.

Practitioners represented numerous behavioral health care disciplines (e.g., clinical psychology, counseling, social work) and had experience working with autistic youth. Eligibility criteria for youth participants included being 6–17 years old, having a clinical autism diagnosis, and engaging in outpatient intervention with a participating practitioner. Other restrictions were minimized to enhance external validity, although participant characteristics were evaluated and recorded. The principal investigator reviewed eligibility, and the study coordinator informed participants of their status. Participants were assigned code numbers to maintain confidentiality.

Practitioners provided services in various settings, including schools, mental health agencies, private practices, and university-based clinics. Background information was collected through open-ended interviews, though not all participants provided complete data. See Table 1 for the practitioners’ demographic information. Table 2 summarizes demographic information for the youth (client) participants.

**Table 1:** Practitioners’ demographic characteristics and experience

Variable	*n* (%)
Female	6/7 (85.7%)
Ethnicity/race	
Latino	1/7 (14.3%)
White	6/7 (85.7%)
Degree/discipline	
Doctorate/psychology	2/7 (28.6%)
Master’s/marriage and family therapy	2/7 (28.6%)
Master’s/social work	1/7 (14.3%)
Masters/other	2/7 (28.6%)
Years as a practitioner	*M* = 8.14, *SD* = 6.23

**Table 2:** Youth client demographic characteristics

Measure	*n* (%)
Female	3/7 (43%)
Ethnicity/race	
Latino	1/7 (14%)
White	5/7 (71%)
Latino and East Indian	1/7 (14%)
Age	*M* = 13.0, *SD* = 2.56

Participant 1 holds an M.A. in Marriage and Family Therapy, is a Board-Certified Behavior Analyst (BCBA), and is a Registered Play Therapist (RPTS). With a primary behavioral orientation, she has worked with 200–300 autistic children, including school consultations. Participant 2 has a Ph.D. in Psychology and extensive experience in CBT, including exposure and response prevention (ERP) and mindfulness-based cognitive therapy. She is licensed and board-certified in CBT. Participant 3 is a pediatric psychologist with a Psy.D. in School Psychology and master’s degrees in clinical counseling and educational psychology. He is a board-certified behavior analyst with a behavioral orientation and 13 years of experience. Participant 4 is a licensed clinical social worker (LCSW) with 6 years of experience and a focus on CBT and Socio-Dramatic, Affective Relational Interventions (SDARI). Participant 5 has a master’s degree in clinical psychology and a background in ABA. She is a licensed marriage and family therapist who has been practicing behavioral and CBT approaches for 5 years. Participant 6 is a clinical psychology graduate student in her third year of doctoral training, with a master’s degree in psychology and experience in CBT and parent management training. Participant 7 is a graduate student with a master’s degree in psychology, experience in ABA, and a theoretical orientation combining CBT and dialectical behavior therapy (DBT).

### Procedures and Randomization

After consent was obtained from practitioners and families and an initial assessment was completed, practitioners were randomized to one of four baseline conditions (2, 5, 6, or 8 sessions). This randomization process, conducted by the last author using a computerized table, ensured impartiality, as this investigator had no direct participant interaction. The study coordinator communicated baseline assignments to practitioners.

#### Baseline Phase

During the baseline phase, practitioners continued their usual psychotherapy or counseling approaches for the designated sessions. The research team recorded sessions using video devices and securely recorded them for later coding.

#### MEYA Phase

Following the baseline period, practitioners received login credentials for the free/open-access MEYA website (www.meya.ucla). They completed a 2-h training video introducing MEYA and incorporated its guidance into their sessions. The MEYA platform provided just-in-time training, offering brief video tutorials on EBPs tailored to the specific needs of each youth. Practitioners were asked to conduct at least eight MEYA-guided sessions unless therapy concluded earlier. Optional consultation calls with a clinical expert were available to address questions about interventions and/or the MEYA platform.

Throughout the MEYA phase, practitioners retained autonomy in session planning and continued video-recording sessions for research purposes. MEYA facilitated a practical approach to learning EBPs, integrating training directly into clinical workflows. MEYA includes six primary clinical focuses: disruptive behavior, negative affect (e.g., anxiety and depression), repetitive/rigid behavior, peer engagement, conversation and friendship, and self-care skills. Modules incorporate content for school- and clinic-based settings, with adaptations for children with minimal expressive language or varying cognitive levels. Practitioner feedback during development ensured usability and relevance. MEYA offers tools like the Session Selector/Planner and the MEYA Chart to streamline intervention planning. The Planner uses caregiver input from an initial assessment of youth’s goals or challenges, scored on a 10-point scale. Weekly updates guide algorithmic recommendations for the most appropriate modules. Practitioners begin with core sessions (e.g., self-management) and progress through clinical areas based on priority ratings or progress achieved.

### Measures

#### MEYA Integrity Scale-Practitioner Version (MEYA-IS-PV)

The MEYA-IS-PV items were designed to parallel the content of the MEYA-FS. Steps taken to establish the content validity of the MEYA-FS items included review of treatment protocols and expert review (see McLeod et al., 2022a for details). The 10 MEYA-FS model items were used for the MEYA-IS-PV: Cognitive, Perspective Taking, Self-Management, Peer Skills, Exposure, In-session Reinforcement, Goal Charts, Home-based Rewards, Positive/Preventative, Parent Training. One MEYA-FS Delivery item was used: Role-Playing. Finally, one MEYA-FS item, Homework, was split into two items for the MEYA-IS-PV: Homework Assigned, Homework Reviewed. This resulted in a total of 13 items. The MEYA-IS-PV items were reworded into a rating form to provide information on how much each practice was used in a specific treatment session. The items were scored on a 5-point scale with the following anchors: 1, *not at all*; 3, *some*; and 5, *a lot*. Hence, higher scores reflect more extensive use of relevant EBPs in the session. This scoring strategy has been used in previous self-report treatment fidelity measures (see Hogue et al., 2014; McLeod et al., 2022b).

After each session (both baseline and MEYA phases), practitioners made self-report ratings on the MEYA-IS-PV according to the following instructions: “Indicate the extent to which you used each technique in today’s session. In making your ratings, please consider both the *frequency* and *thoroughness* with which you used each technique. For any techniques that you did not use, please circle ‘1’ for ‘not at all.’” Sample items include the following: “Taught or encouraged the child to identify others’ perspectives,” “Encouraged the child to participate in exposure(s) to specific challenging situations to build emotion-related coping skills and reduce target symptoms,” “Taught or encouraged parents to use rewards and privileges at home to achieve treatment goals,” and “Taught or encouraged child to practice positive peer relationship skills such as conversations, joining games, sharing, or hosting playdates in session and/or at home.”

#### Modular EBPs for Youth on the Autism Spectrum Fidelity Scale (MEYA-FS)

As detailed in McLeod et al. (2022a) and Wood et al. (2024), the MEYA-FS is a 16-item measure designed to evaluate practitioner adherence and competence in EBPs for autistic youth. Parallel adherence and competence items assess universal elements common to most evidence-based programs (e.g., assigning out-of-session tasks) and core practices (e.g., perspective-taking training, exposure) related to CBT and BI practices for autistic youth. Coders review session recordings and rate adherence on a 7-point scale from 1 (“not at all”) to 7 (“extensively”).

The MEYA-FS Change in Adherence and Competence subscales track practitioners’ improvement in applying EBPs within modular interventions. These subscales estimate fidelity regardless of the modules delivered. Scores are derived from four item clusters: common EBP practices (e.g., modeling, reinforcement), social-communication components (e.g., perspective-taking), behavioral procedures (e.g., self-management, exposure), and generalization strategies (e.g., homework, goal charts). The present study used MEYA-FS Change in Adherence scores to compare with practitioner self-reports on the MEYA-IS-PV (below), which focuses on adherence (not competence). Higher subscale scores reflect greater adherence to autism-related EBPs.

Three doctoral students made MEYA-FS ratings after a systematic training phase (see Wood et al., 2024, for details). Training with the last author entailed didactic instruction, a review of the scoring manual, session discussions, and coding exercises. To be certified for independent coding, raters met reliability criteria for each item on 30 training tapes. Each session was double-coded, and raters were naïve to study hypotheses. As Wood et al. (2024) reported, inter-rater reliability was ICC (2,2) = .79.

#### Youth Characteristics

Baseline assessments were used to evaluate child/youth clinical and developmental profiles. The Social Responsiveness Scale 2 (SRS-2; Constantino & Gruber, 2012), a 65-item parent-reported scale, measured autism-related challenges like social awareness and preoccupations. This tool is reliable and distinguishes children on the autism spectrum from non-autistic children. To assess oral language abilities, youth completed the Vocabulary subtest of the WISC-V, a validated ability test for youth aged 6 to 16 (Wechsler, 2014), over the phone. All participants were native English speakers.

#### Youth Clinical Needs

The Youth Top Problems scale (YTP), a personalized symptom assessment sensitive to treatment effects in autistic youth (Weisz et al., 2011; Wood et al., 2022), was used at baseline. Parents identified the most pressing issues within six clinical areas during a semi-structured interview and rated severity on a 0–10 scale. Weekly ratings of up to 12 prioritized problems or goals provided data for personalized session planning as well as assessment of treatment outcomes.

### Data Analysis

Psychometric analyses of practitioner self-reports on the MEYA-IS-PV addressed inter-item correlations, convergence with expert ratings on the MEYA-FS, and two indices of concurrent validity: responsiveness to training (within the context of the multiple baseline design) and association with youth outcome (on YTP ratings). Linear mixed modeling (LMM), which accounts for the patterns of nesting in this study (e.g., sessions nested within practitioners), was used to test hypotheses pertaining to convergent and concurrent validity using SPSS software (Version 29). LMM is a full-information analytic procedure that estimates the treatment effect in multiple baseline studies using the Satterthwaite method coupled with an autoregressive level 1 error structure (Ferron et al., 2009). Visual analyses and phase-related nonoverlapping frequency tabulations of the multiple baseline practitioner self-report data were also conducted.

## Results

Descriptive statistics for the MEYA-IS-PV, MEYA-FS, and YTP scores are as follows: Ms (SDs) = 32.99 (9.09), .86 (.76), and 7.14 (2.06), respectively. The average inter-item correlation for the MEYA-IS-PV was .22, and the squared multiple correlation between each item and the scale total ranged from .39 to .75. Hence, the 13 items were considered to work together adequately as a scale for hypothesis testing. To examine convergent validity, an LMM was estimated in which practitioner-rated MEYA-IS-PV scores were used to predict expert adherence ratings on the MEYA-FS. In this two-level model, sessions were nested within practitioners, and a random intercept was included. The parameters for this model are presented in Table 3. This model had a statistically significant fixed effect for practitioner ratings, with a corresponding pseudo-*R*^2^ (for the fixed effects) of .30, representing a moderate degree of correspondence (i.e., equivalent to *r* = .55, categorized as a large effect). The conditional ICC for practitioner effects in this model was .38.

**Table 3:** LMM model for MEYA-IS-PV scores predicting expert ratings of practitioner adherence on the MEYA-FS

MEYA-FS change in adherence				
Fixed effect	Coefficient	Standard error	*t*-ratio	*p-*value
Intercept	−.96	.358	−2.69	.012
MEYA-IS-PV scores	.06	.008	6.62	< .001
Random effect	Variance component	Standard error		
Residual	.27	.05		
Intercept (practitioner)	.33	.21		

*Note.* *N* = 7

**Fig. 1:**
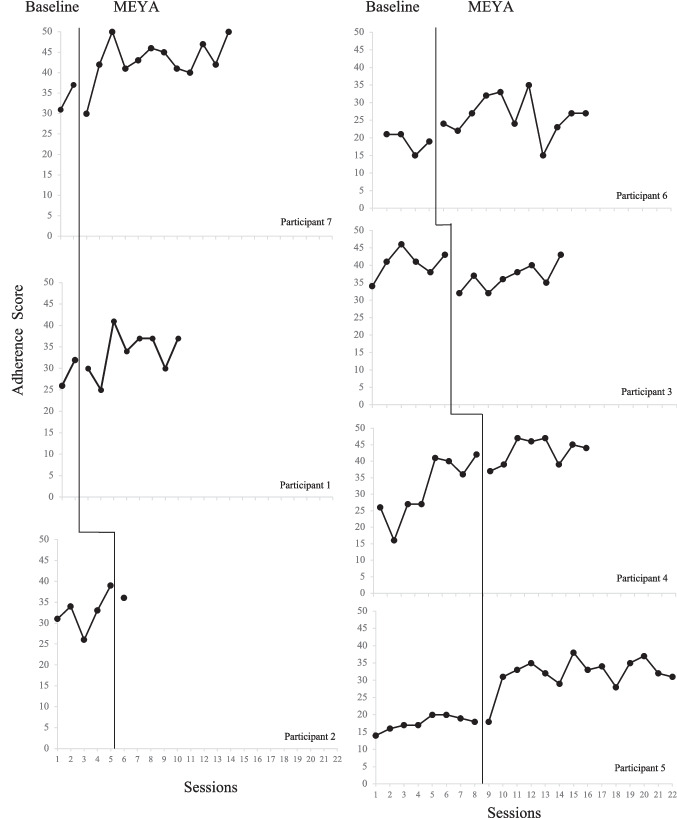
Self-reported adherence to evidence-based practices

Visual analysis shows that five of the seven participants increased their adherence to EBPs covered in MEYA, with nonoverlapping data in the majority of sessions (Participants 1, 4, 5, 6, and 7: 62.5%, 62.5%, 93.3%, 90.9%, and 91.7%, respectively) following the onset of their access to the MEYA website (see Fig. 1), in comparison to their highest MEYA-IS-PV score during the baseline period. It is important to note there is variability in the timing and magnitude of change for these participants, as shown in Fig. 1. Conversely, two of the seven participants (Participants 2 and 3) had largely overlapping data across phases for their MEYA-IS-PV scores. Both participants had very high baseline scores and little room to improve on this scale, one of whom terminated after only one recorded session post baseline due to unknown reasons.

An LMM analysis was conducted to examine the impact of MEYA training on MEYA-IS-PV scores. The initial model, described in the “Data Analysis” section, failed to converge due to an indefinite Hessian matrix but indicated a statistically significant treatment effect. After removing the random treatment effect, the simplified model converged successfully. Coefficients from this model, summarized in Table 4, demonstrated a significant positive effect for phase, where MEYA-IS-PV scores increased after practitioners began using the MEYA website. This result aligns with the graphical data presented above, supporting the interpretation that MEYA training positively influenced practitioner adherence.

**Table 4:** LMM model for effect of MEYA versus baseline on MEYA-IS-PV scores

*MEYA-IS-PV scores*				
Fixed Effect	Coefficient	Standard Error	*t*-ratio	*p-*value
Intercept	28.81	2.66	7.38	<.001
MEYA	7.77	1.34	5.78	<.001
Random Effect	Variance Component	Standard Error		
Residual	34.27	5.25		
Intercept (practitioner)	42.04	25.69		

*Note.* N=7. The fixed effect of MEYA represents the increase in MEYA-IS-PV scores once access to MEYA training was provided to a participant following their baseline phase

To examine youth outcomes on the YTP measure, a 3-level LMM was estimated in which individual YTP items were nested within sessions, which were nested within therapists. Random intercepts for levels 2 and 3 of this model were included. Fixed effects at level 2 of the model included the YTP clinical area (modeled as a 6-category IV, representing the following clinical areas: disruptive behavior, negative affect, repetitive/rigid behavior, peer engagement, conversation and friendship, and self-care skills) and MEYA-IS-PV scores at each session. The interaction between these two fixed effects was also modeled. The Type III tests of fixed effects and random effects are presented in Table 5. To further elucidate the fixed effects, statistically significant estimates of fixed effects are noted here: MEYA-IS-PV scores had a negative association with YTP scores, such that higher EBP adherence was associated with lower youth behavioral health problem scores (*b* = −.08, SE = .06, *p* = .002); the YTP friendship and conversation category had a lower intercept than other categories (*b* = −1.60, SE = .70, *p* = .023); and there was an interaction between MEYA-IS-PV scores and disruptive behavior, such that higher MEYA-IS-PV scores had a significantly greater positive effect (lower YTP) scores on YTP items related to disruptive behavior than on other categories of youth behavioral health need (*b* = −.05, SE = .02, *p* = .042). Overall, greater practitioner adherence to MEYA practices was associated with greater improvement in youth behavioral health.

**Table 5:** Three-level LMM model for effect of MEYA-IS-PV scores on YTP scores (nested within practitioner and session)

Type III tests of fixed effects				
Source	Numerator df	Denominator df	*F*	*p-*value
Intercept	1	27.34	156.01	< .001
MEYA-IS-PV scores	1	41.73	18.18	< .001
YTP category	5	410.30	2.10	.064
MEYA-IS-PV × YTP category	5	412.03	2.58	.026
Random effect	Variance component	Standard error		
Residual	1.68	.12		
Intercept (practitioner)	1.15	.74		
Intercept (practitioner × session)	.59	.18		

*Note.* *N* = 7. The YTP category variable is a 6-category IV, representing the following clinical areas: disruptive behavior, negative affect, repetitive/rigid behavior, peer engagement, conversation and friendship, and self-care skills

## Discussion

Practitioner report adherence measures can support effectiveness and implementation research. This study investigated the initial score validity of a brief, practitioner-rated adherence measure. These findings suggest that scores on practitioner self-rating of adherence to EBPs for autistic youth in psychotherapy or counseling display evidence of validity related to applications in effectiveness and implementation research on practices drawn from CBT and behavioral intervention paradigms. Specifically, evidence of inter-item correspondence, convergence with expert adherence ratings, responsiveness to training in EBPs, and positive association with youth outcomes (rated by a separate informant) contributed to an overall positive initial psychometric profile for the MEYA-IS-PV scale.

From the standpoint of convergent validity, this study noted the relative concurrence between practitioners’ ratings of their in-session practices and the observational ratings of independent coders unaware of phase (baseline or MEYA) on adherence to EBPs. The size of the concurrence (large) is considerably higher than what is typically observed between practitioner and observer ratings of adherence (see McLeod et al., 2023 for a review). Evidence of convergence is a foundational component in establishing a measure within the nomological network (Cronbach & Meehl, 1955), and the entirely independent nature of the ratings from two measurement sources bolsters confidence in the observed association between the scores. The size of the association indicates that scores on the MEYA-IS-PV can be interpreted as adherence to EBPs.

An important element in a treatment adherence measure used in effectiveness and implementation research is its responsiveness to changes in practices. Our primary implementation outcome study (Wood et al., 2024) clearly illustrated that expert ratings of adherence to EBPs measurably increased among five of the seven participating practitioners once practitioners began training in EBPs and attempted to implement the EBPs in their psychotherapy or counseling sessions. The same five participants provided a pattern of adherence self-ratings that mirrored the expert ratings’ data; once training in EBPs began, visual analysis showed their self-ratings of adherence increased. Conversely, a minority of participants (2 of 7) did not show this pattern. However, their pre-training adherence levels were high, likely reflecting a general evidence-based therapeutic style. The fact that the practitioner- and observer-rated measures demonstrated the same pattern indicates that each demonstrates sensitivity to change over time, albeit at varying magnitudes. This means that the measures can be used to gauge the success of training and supervision on practitioner adherence, an important feature for treatment adherence measures used in effectiveness and implementation research (Sutherland et al., 2022).

This finding underscores an important feature of most extant EBPs: they are often practices that many practitioners encounter (in some form) in their pre-service training and sometimes adopt in their professional work. There are also many other practices in psychotherapy and counseling that have less evidentiary support that most practitioners in community practice also often employ (Pickard et al., 2018). Many EBPs utilized in programs like MEYA are common techniques (e.g., exposure, reward, social skills training) largely tailored to a specific population (e.g., age group, clinical needs) and integrated across sessions. Therefore, it is not entirely surprising to encounter practitioners already utilizing a relatively high proportion of relevant EBPs in their practice before engaging in additional training. Further inquiry is warranted to better understand the impact of training for practitioners of varying experience and training.

Arguably, the sine qua non of an EBP adherence measure is its association with clinical outcomes. For established EBPs that outperform other practices for a given clinical need in randomized, controlled trials, it is nearly self-defining that the greater the adherence to such practices, the better the youth’s outcomes. This pattern of findings was previously established for the expert-rating version of this adherence measure (i.e., the MEYA-FS) in its initial psychometric study (McLeod et al., 2022a). Notably, in the present study, practitioners’ ratings of their own adherence to relevant EBPs were also associated with improvements in youth behavioral health on a personalized measure of youth clinical needs rated at each session by their caregivers (the YTP). In the context of the evidence of reliability and convergent validity summarized above, this additional finding suggests that the estimates of EBP utilization derived from the MEYA-IS-PV may have practical significance (for youth outcomes) beyond its promising measurement precision.

Despite these positive outcomes from this study, several weaknesses exist. The small sample size limits the generalization of the findings. Additional research with a larger sample that is representative of a diverse group of practitioners and youth from the perspective of race and ethnicity, gender, practitioner discipline and background training, clinical setting, and youth communication skills would be helpful in better understanding how the measure performs in important varying contexts. In spite of these limitations, the study was characterized by several strengths, including its single-case experimental design, repeated measures, multiple informants, and the use of mixed modeling, which takes advantage of these methodological strengths. This study offers a promising initial signal that practitioner self-reports of EBP’s use in psychotherapy or counseling for autistic youth can be a valid and reliable tool for implementation research as well as for possible use in clinical settings in which stakeholders are looking to support youth outcomes by encouraging and monitoring the use of effective practices.

## Data Availability

Data is available upon request.
